# Goji Berry Intake Increases Macular Pigment Optical Density in Healthy Adults: A Randomized Pilot Trial

**DOI:** 10.3390/nu13124409

**Published:** 2021-12-09

**Authors:** Xiang Li, Roberta R. Holt, Carl L. Keen, Lawrence S. Morse, Glenn Yiu, Robert M. Hackman

**Affiliations:** 1 Department of Nutrition, UC Davis, Davis, CA 95616, USA; xxlli@ucdavis.edu (X.L.); rrholt@ucdavis.edu (R.R.H.); clkeen@ucdavis.edu (C.L.K.); 2 Department of Internal Medicine, UC Davis, Sacramento, CA 95817, USA; 3 Department of Ophthalmology and Vision Science, UC Davis Medical Center, Sacramento, CA 95817, USA; lsmorse@ucdavis.edu (L.S.M.); gyiu@ucdavis.edu (G.Y.)

**Keywords:** goji berry, zeaxanthin, lutein, carotenoids, age-related macular degeneration, macular pigment optical density

## Abstract

Age-related macular degeneration (AMD) is the third leading cause of blindness worldwide. Macular pigment optical density (MPOD), a biomarker for AMD, is a non-invasive measure to assess risk. The macula xanthophyll pigments lutein (L) and zeaxanthin (Z) protect against blue light and provide oxidant defense, which can be indexed by MPOD. This study examined the effects of Z-rich goji berry intake on MPOD and skin carotenoids in healthy individuals. A randomized, unmasked, parallel-arm study was conducted with 27 participants, aged 45–65, who consumed either 28 g of goji berries or a supplement containing 6 mg L and 4 mg Z (LZ), five times weekly for 90 days. After 90 days, MPOD was significantly increased in the goji berry group at 0.25 and 1.75 retinal eccentricities (*p* = 0.029 and *p* = 0.044, respectively), while no changes were noted in the LZ group. Skin carotenoids were significantly increased in the goji berry group at day 45 (*p* = 0.025) and day 90 (*p* = 0.006), but not in the LZ group. Regular intake of goji berries in a healthy middle-aged population increases MPOD may help prevent or delay the development of AMD.

## 1. Introduction

Age-related macular degeneration (AMD) is the leading cause of blindness among seniors in developed countries, and third worldwide after uncorrected refractive errors and cataracts [1,2]. In early stages, the disease is characterized by small to intermediate drusen with pigmentary changes that may progress rapidly to more advanced forms such as choroidal neovascularization or central geographic atrophy with loss of central vision [3]. Lutein (L), zeaxanthin (Z), and the isomer meso-zeaxanthin (meso-Z) are macular pigments that filter damaging blue light and provide oxidative defense in the macula. These pigments are found in plants as xanthophylls, with increased dietary intake proposed to reduce the development and progression of AMD [4]. The relative concentration of xanthophyll carotenoids in the retina can be measured non-invasively by psychophysical and objective methods, expressed as macular pigment optical density (MPOD) [5]. Numerous epidemiological studies report that individuals with a low MPOD level are at an increased risk of AMD [6].

Dietary L and Z are found in certain fruits and vegetables with red, yellow, or orange color, egg yolk, and in some green leafy vegetables [7,8]. The dietary intake of Z is lower than L in all age groups and ethnicities in the U.S. [9]. Dietary intakes of L and Z are strongly associated with their serum levels, as well as with MPOD [10]. Previous studies have shown that high intakes of these carotenoids from dietary sources or supplements can increase plasma L and Z, and MPOD [11]. Once early AMD has progressed to the intermediate stage, dietary supplements are indicated, but no clinical evidence yet exists for interventions that can address the prevention of small-intermediate drusen with pigmentary changes, the initial clinical signs of macular disruption [12].

Goji berry (*Lycium barbarum* L. and *L. chinense*), also termed wolfberry or Go Chi Zi, has been used in traditional Chinese medicine for more than 2000 years [13]. The bright red berry contains the highest amount of Z among all known dietary sources and is mainly present in a dipalmitate form [14,15]. The intake of zeaxanthin dipalmitate (ZD) extracts from goji berry increases plasma Z to a greater extent than non-esterified Z supplementation [16]. The berries also contain unique carbohydrates that are present as conjugates with peptides or proteins, which are often referred to *L. barbarum* polysaccharides (LBP). These have shown anti-inflammatory and neuroprotective effects in animal and cell culture studies [17].

The typical adult human eye has approximately 2.4 times more Z than L in the central fovea of the macula [18], making goji berry intake a prime candidate for increasing MPOD. Nevertheless, there is a paucity of clinical evidence on goji berry and MPOD particularly for the prevention or delay of progression from early to intermediate AMD. In individuals from China with signs of early AMD, 25 g of daily consumption of goji berries for 90 days significantly increased both serum Z and MPOD [19]. However, this study had a broad age range (51 to 92 years of age), some participants smoked, and others had certain pre-existing medical conditions. Additionally, the authors only reported central MPOD values up to 0.5 retinal eccentricity (RE), whereas macular pathology and visual dysfunction in AMD may extend beyond that central region. Therefore, to provide a more complete understanding of the influence of goji berry intake on the progression AMD, data is needed on for different population groups that measures MPOD at eccentricities over the entirety of the macula.

In the current study, we prospectively evaluated if the daily intake of 28 g of goji berries or a commercially available supplement providing 6 mg of L and 4 mg for 90 days can improve MPOD and skin carotenoid levels, an index of total carotenoid intake, among healthy middle-aged adults, 45 to 65 years old, with no signs of drusen or early AMD.

## 2. Materials and Methods

### 2.1. Participants

Eighty-eight volunteers, ages from 45 to 65 years old, were recruited from an online website and public advertisements in the area of greater Sacramento, California. Participants provided informed consent and were screened with a questionnaire. Inclusion criteria were being generally healthy (not currently under medical supervision, free from self-reported diabetes, cancer, heart, kidney or liver diseases and gastrointestinal disorders), having a normal macular condition as verified by an optometrist, and if relevant, being prescribed the same medication regimen for at least 6 months that was not related to carotenoid metabolism and was approved by the study physician. Exclusion criteria were a dislike of, or allergy to goji berries, diseases of the eye, malabsorption problems, substance or alcohol abuse, smoking, drugs for management of lipids, glucose, or blood pressure, use of dietary supplements other than multivitamins and minerals that provided greater than 100% of the U.S. Dietary Reference Intake, or any supplement containing L or Z. The intervention was registered on ClinicalTrials.gov (NCT03983525) (accessed on 21 July 2020), with the first posted date of 6 December 2019, complied with the tenets of the Declaration of Helsinki, was approved by the Institutional Review Board of the University of California (UC), Davis (IRB #1220178) and was conducted at the UC Davis Ragle Human Nutrition Research Center.

### 2.2. Study Design

Qualified participants were randomized into a prospective, parallel-arm, unmasked study to consume either 28 g of goji berries or a commercially available supplement of L and Z five days per week for 90 days. Study measurements were collected at baseline (prior to supplement or goji berry intake; day 0), at 45 ± 2 days and 90 ± 2 days after intake.

Twenty-eight grams of goji berries is considered a single serving size [20]. The berries in this study were USDA-certified organic goji berries grown in the Ningxia region of northern China and provided by Navitas Organics, Novato CA, USA. The goji berries were portioned into clean, single-serving plastic bags and provided in 45-day allotments. The commercially available supplements (Source Naturals, Scotts Valley, CA, USA, lot #FG-91753) were purchased online, contained 6 mg of L and 4 mg of Z per serving and were repackaged into 45-day supplies in clean plastic bottles. Compliance was monitored by a self-administered log. Habitual dietary information was collected with the Automated Self-Administered 24 h dietary assessment web-based tool (ASA24; https://epi.grants.cancer.gov/asa24, accessed on 10 August 2020) once between day 0 and 45, and once again between day 45 and 90.

The MPOD was assessed by the psychophysical method of customized heterochromatic flicker photometry using a macular densitometer (Macular Metrics, Providence, RI, USA). After participants viewed a 5-minute video detailing the measurement procedures, they were dark-adapted for 7 minutes and then began the test. The light intensity of each relevant wavelength was calibrated with a photodiode. The flicker frequency was selected based on a preliminary test of the participant’s sensitivity. The task was to eliminate or minimize the flicker in the visual field three times by turning a dial that changed the intensity of a 460 nm light. Each participant performed the test while looking directly at the flickering light at 0.25, 0.5, 1, and 1.75 RE degrees, representing the MPOD level from the center to the periphery of the macula.

Skin carotenoid content was measured by reflection spectroscopy (“Veggie Meter”, Longevity Link Corporation, Salt Lake City, UT, USA). After cleaning, the tip of the right index finger was inserted into the spectrophotometer and three measurements were collected. A skin carotenoid score was calculated by the system software. Carotenoids that exist in human plasma, including β-carotene, lycopene, L, Z, and their isomers have been successfully detected in toto and quantified by this device [21,22], which has been validated to reflect fruit and vegetable consumption [23].

### 2.3. Statistical Analysis

Sample size was based on a study that assessed the impact of a Z supplement on MPOD in 24 healthy people [24]. Statistical analyses were performed with JMP version 16 (SAS Institute Inc., Cary, NC, USA). Two-tailed *t*-tests evaluated potential between-group differences at baseline. The MPOD and skin carotenoid data were analyzed with mixed-effects models using time and treatment as the main factors, with age and sex as the covariates, and participant ID as the random effect. For main effects, student *t*-tests determined significance within group pairs. *p*-Values of 0.05 or less were considered statistically significant. Correlation coefficients between the outcome measures were determined via Spearman’s method. The mean values of the dietary intake data were compared by two-tailed *t*-tests, which were log-transformed when necessary, and presented as the mean ± S.E.M. or the back-transformed mean with 95% confidence intervals (CI).

## 3. Results

**Figure 1:**
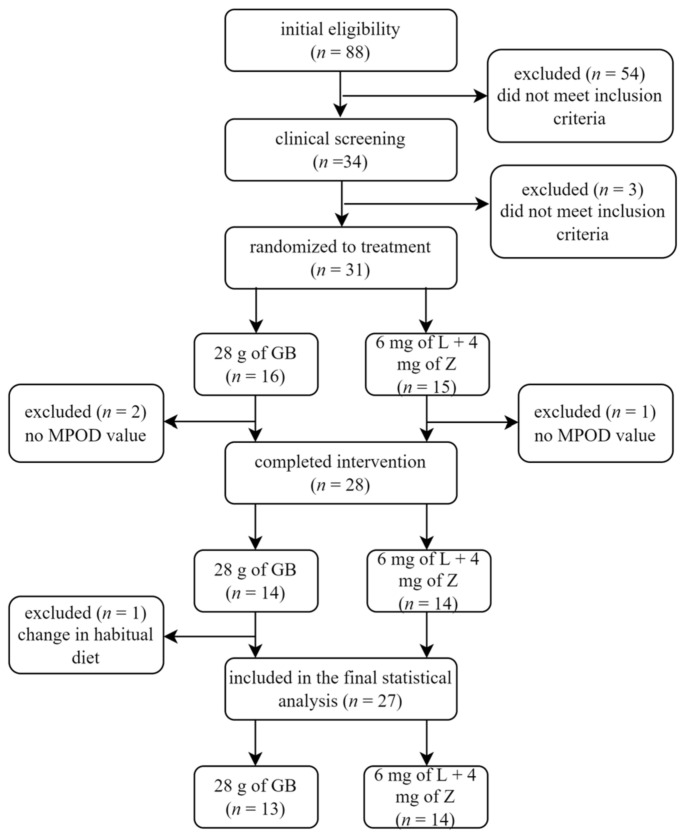
Participant flow diagram. Thirty-one participants were randomly assigned to consume either 28 g of goji berries (GB) or a supplement containing 6 mg of lutein (L) and 4 mg of zeaxanthin (Z), five times per week for 90 days. Twenty-eight individuals completed the study. An *n* = 13 in the GB group and an *n* = 14 in the LZ group were used in the statistical analysis.

Thirty-one healthy, middle-aged adult males and females (mean age of 56 years) met the inclusion criteria between May 2019 and Jan 2020. The participants consumed either goji berries (*n* = 16) or the LZ supplement (*n* = 15) 5 days per week for 90 days. Twenty-eight individuals completed the intervention, after which two in the goji berry and one from the LZ group were excluded from the data analysis due to measurement errors. Furthermore, data from one was subsequently removed after learning of a major change in dietary patterns that included a low intake of macronutrients between days 45 and 90 (Figure 1).

Reported protocol compliance was greater than 96% for both groups, and no adverse symptoms were noted other than minor intestinal gas from one participant in the goji berry group. Table 1 presents the reported average intake of select nutrients in the habitual diet that may have affected eye health over the study period. No significant differences between groups were noted. The composition of the goji berries is presented in Table 2. A daily goji berry serving provided 28.8 mg of Z, which was substantially higher than the 4 mg of Z present in the supplement. Although sufficient extraction of L from our goji berry samples could not be obtained, previous work by others estimated a L content of 0.15 mg in 28 g of goji berries from six different goji berry samples collected in the Ningxia province of China, the same region from which the goji berries used in this study were obtained [25].

**Figure 2:**
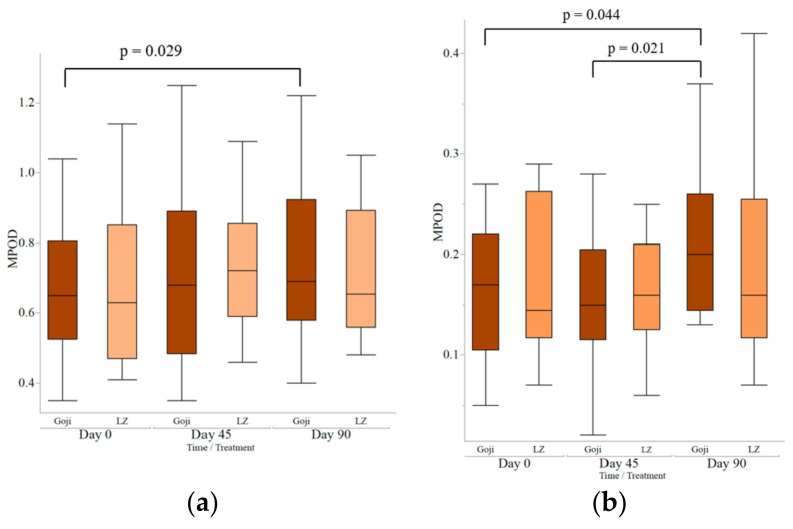
(**a**) Three months of goji berry intake increased macular pigment optical density (MPOD) at 0.25 retinal eccentricity (RE) degrees, at Day 90 compared to baseline (Day 0) and at day 45. (**b**) Three months of goji berry intake increased macular pigment optical density (MPOD) at 1.75 retinal eccentricity (RE) degrees, at Day 90 compared to baseline and at day 45. Statistical analysis performed by mixed models using time and treatment as the main factors, and age and sex as the covariates with Student’s *t*-test for pairwise comparisons; boxplots are the median and interquartile range.

Baseline MPOD measures were similar between the goji berry and supplement groups (Table 3). No significant interaction effects for treatment and time were observed in any REs. A significant main effect of time was found for MPOD at 0.25 RE (*p* = 0.023). In a sub-analysis, intake of goji berries, but not LZ, significantly increased MPOD at 0.25 RE at day 90 compared to baseline (*p* = 0.029; Figure 2a). There was also a significant main effect of time for MPOD at 1.75 RE (*p* = 0.039), with a significant increase at day 45 compared to the baseline (*p* = 0.021), and again between day 90 (*p* = 0.044; Figure 2b). No significant MPOD changes were noted at any REs in the LZ group.

**Figure 3:**
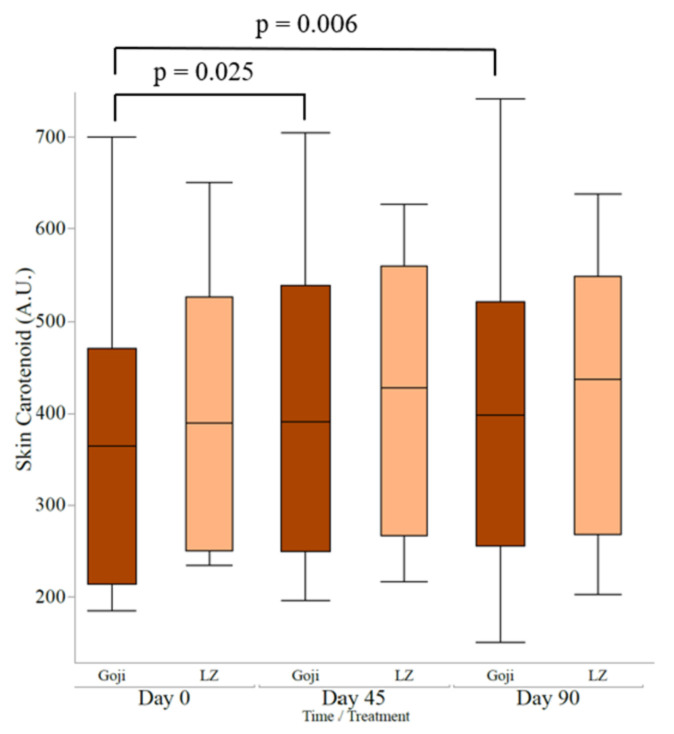
Three months of goji berry intake increased skin carotenoid score at Day 45 and Day 90 compared to Day 0. No changes in the lutein and zeaxanthin supplement (LZ) group were noted. Statistical analysis performed by mixed models using time and treatment as the main factors, and age and sex as the covariates with student *t*-test for pairwise comparisons; boxplots are the median and interquartile range.

Baseline skin carotenoid scores were not significantly different between the goji berry and LZ groups (Table 3). No significant interaction effects for treatment and time were observed. However, the main effect of time was significant (*p* = 0.011). This corresponded to a significant increase from baseline after 45 and 90 days in the goji berry group (*p* = 0.025 and 0.006, respectively), while no significant changes were noted in the LZ group (Figure 3). The absolute values of MPOD and skin carotenoid scores are shown in the Supplementary Table S1.

Overall, skin carotenoid scores were significantly correlated with MPOD at 0.25 (ρ = 0.33, *p* = 0.004), 0.5 (ρ = 0.41, *p* = 0.0002), and 1 RE (ρ = 0.38, *p* = 0.0007; Supplementary Figure S1.1). The skin carotenoid score was not correlated with MPOD at any of the REs for the goji berry group (Supplementary Figure S1.2). In contrast, for the LZ group, the skin carotenoid score was significantly correlated with MPOD at 0.25, 0.5, and 1 RE (0.25 RE: ρ = 0.55, *p* = 0.0003; 0.5 RE: ρ = 0.57, *p* = 0.0002; 1 RE: ρ = 0.54, *p* = 0.0004), with a trend at 1.75 RE (ρ = 0.31, *p* = 0.06; Supplementary Figure S1.3).

## 4. Discussion

Ninety days of 28 g of goji berry intake significantly increased the optical biomarker MPOD in healthy adults at 0.25 and 1.75 REs. These results suggest that even in a healthy population with no evidence of small drusen or early AMD, goji berry intake can improve eye health. Our results are consistent with data of improved MPOD after a similar amount and intake period of goji berry in a Chinese population at risk for intermediate AMD [19]. Moreover, our trial is consistent with reports of protection against macular hypopigmentation and drusen development in a population of generally healthy and older (65 to 75 years of age) individuals who were provided Z at approximately a third of the amount of Z provided in the current trial (i.e., 10 mg/d of Z derived from goji berries) [26]. Our findings suggest that a higher intake of Z relative to L may be useful in reducing the risk of AMD. This is consistent with increased MPOD levels after 4 months of supplementation with 20 mg Z or 26 mg Z with 8 mg L plus 190 mg of mixed omega-3 fatty acids by young healthy adults [27]. Interestingly, we observed a significant increase in MPOD at 1.75 RE, but not at 0.5 or 1 RE, in the goji berry group. A possible explanation for this trend is the relatively low macular pigment at 1.75 RE compared to the other REs, which may increase the potential for improved MPOD in this peripheral area of the macula. Our results are also consistent with data from 11 randomized controlled trials where supplementation with at least 10 mg of the macular carotenoids was effective at increasing MPOD [28].

Significant correlations were observed between the overall skin carotenoid score and MPOD, which is consistent with clinical results of carotenoid supplementation [29]. Further analysis demonstrated that L and Z, but not goji berry intake, was significantly influencing this trend. Previous work has shown an association between serum L and Z in skin and blood with macular pigment carotenoid accumulation [29]. Data from the current trial are consistent with this observation as goji berry intake was significantly associated with the skin carotenoid score. However, in contrast to data with L and Z supplements, MPOD score was not correlated with changes in skin carotenoids with goji berry intake. The skin photometer detects overall carotenoid content, and as goji berries are also rich in β-carotene, neoxanthin, and cryptoxanthin [30], these carotenoids likely influenced the skin measurements, and would not reflect the selective carotenoid accumulation of L and Z in the macula. Other goji berry components such as taurine, vitamin C, zinc, and LBP may influence the results by lowering oxidant stress and improving eye health [31,32,33]. For example, studies in animals and cell lines suggest that LBP can protect against AMD by reducing oxidative stress and cell apoptosis in retinal pigment epithelium [34]. Taken together, under the conditions tested, it is reasonable that MPOD may not fully correlate with skin carotenoids in the goji berry group.

To our knowledge, the impact of goji berry intake on MPOD in healthy middle-aged people has not been previously reported. While others have noted improved MPOD after LZ supplementation among people with low MPOD baseline levels [35], our findings suggest that even in populations with normal MPOD values, a significant increase can be detected after goji berry consumption at the most central part of the macula (0.25 RE). A meta-analysis regarding the effects of L, Z, and meso-Z supplementation noted that the MPOD at baseline was inversely associated with macular responses, suggesting individuals with a relatively lower macular pigment status may receive more benefit with higher amounts of L or Z [36].

The Age-Related Eye Disease Study 2 (AREDS2) trial assessed the impact of dietary supplements containing 10 mg of L, 2 mg of Z, 500 mg of vitamin C, 400 IU of vitamin E, 80 or 25 mg of zinc, 2 mg of copper, and/or 350 mg of docosahexaenoic acid plus 650 mg of eicosapentaenoic acid [37]. The results showed a significantly reduced rate of progression from intermediate- to late-stage AMD after 5 years [38,39]. Secondary analyses of the study indicated protective roles of L and Z [38]. We did not use the AREDS2 supplement for the comparison group because this formula has only been shown to be effective for those with intermediate AMD [39], and no clinical evidence exists for its efficacy in our study population of healthy people. In addition, we note that 80 mg of zinc in the AREDS2 supplement is twice the upper limit of recommended daily intakes for zinc [40].

In epidemiological studies, L and Z intakes have been inversely associated with the development of AMD [37,41]. In the current study, the reported dietary intake of L plus Z, not including the berries or supplement, was 3.1 and 1.9 mg/d in the goji berry and supplement groups, respectively, which is higher than the typical estimated intakes in the US of 1.6–1.86 mg/d [42]. Three to five mg/d of L and Z have been recommended to help support normal macular function, although no recommended dietary allowance values yet exist [8].

A few studies have explored the effects of L and Z from a whole food on MPOD. Daily consumption of one Hass avocado containing 0.5 mg of L over 6 months was associated with a significant increase in MPOD in healthy adults [43]. In contrast, no increase in MPOD was observed after consuming one Hass avocado daily for 3 months [44]. Daily consumption of egg yolks providing 1.38 mg L and 0.21 mg Z resulted in a significant increase in MPOD and other measures of visual acuity in older adults with signs of early-stage AMD after 12 months [45]. Another study giving older adults two egg yolks/day for 5 weeks, followed by four egg yolks/day for 5 weeks, reported increases in MPOD, but only among those with low baseline MPOD values [46]. The addition of either spinach (10 mg L, 0.3 mg Z) or corn (0.4 mg L, 0.3 mg Z), or the combination, for 14 months significantly increased the MPOD among the majority of healthy individuals [47].

Our study has some limitations. Choice of a control is always a challenge in whole food studies, since masking is an issue. A commercially available LZ supplement was used, rather than an inert capsule, since our research design was intended to compare options available to consumers and explore the role of goji berries over and above the intake of purified L and Z. The actual amount of L and Z in the supplement was not confirmed. A previous report noted that the carotenoid content of some powder-based supplements tested in 2017 did not meet label claims, while oil-based supplements did [48]. Since L and Z are preferentially deposited at different eccentricities in the retina, the different amounts of Z in the goji berries and supplement may not be ideal. Volunteers were not screened for low MPOD as an inclusion criterion. Although the relatively modest number of participants in each group may raise some concerns, these numbers are similar to those reported by Obana et al. and are consistent with an initial probe study [49]. Finally, although MPOD was the primary outcome measure, other ocular measurements such as contrast sensitivity and best corrected visual acuity were not assessed. Future studies on goji berry intake and eye health ideally should combine functional and anatomic measurements.

## 5. Conclusions

In conclusion, this study shows that 90 days of goji berry consumption was associated with an increase in MPOD in healthy, middle-aged adults. In addition to L and Z, other bioactive compounds in goji berries may be involved in the increase in MPOD. Further research on goji berries is warranted as both a dietary strategy to reduce the risk of AMD and to serve as part of an integrative approach to mitigate the consequences of this disorder.

## Figures and Tables

**Table 1:** Mean intake of select dietary nutrients, apart from intake of goji berries (GB) or lutein and zeaxanthin (LZ) supplementation, collected once between day 0 and day 45, and again between day 45 and day 90.

	GB	LZ	*p*-Value
Energy (kcal)	2146.4 ± 187.7	1984.3 ± 151.5	0.51
Protein (g)	89.3 ± 10.1	72.7 ± 7.2	0.18
Total Fat (g)	84.2 ± 9.3	84.4 ± 8.8	0.98
Carbohydrate (g)	256 ± 26	241 ± 17	0.6
Vitamin A (mcg)	807.8 ± 120.6	578.3 ± 58.4	0.07
Vitamin C (mg)	120.0 ± 18	103.9 ± 13.8	0.48
Vitamin E (mg) ^1^	14.4 (8.6, 24.1)	11.0 (9.0, 13.3)	0.21
Zinc (mg)	11.8 ± 0.8	9.5 ± 0.9	0.08
Retinol (mcg)	307.1 ± 51.5	265.4 ± 40.5	0.52
β-carotene (mcg)	5127.6 ± 874.0	3408.1 ± 680.4	0.13
α-carotene (mcg) ^1^	300.3 (81.3, 1109.0)	205.9 (87.7, 483.4)	0.58
β-cryptoxanthin (mcg) ^1^	156.9 (33.6, 732.2)	91.0 (53.1, 156.0)	0.4
Lycopene (mg) ^1^	7.2 (3.1, 15.0)	3.6 (1.7, 7.3)	0.2
Lutein + zeaxanthin (mg) ^1^	3.1 (1.7, 5.5)	1.9 (1.1, 3.2)	0.2
DHA (g) ^1^	44.9 (12.9, 156.4)	37.0 (15.8, 86.5)	0.77
DPA (g) ^1^	16.2 (7.1, 36.6)	9.3 (5.1, 16.9)	0.23
EPA (g) ^1^	11.8 (2.5, 56.5)	13.5 (6.0, 30.2)	0.86

Statistical analysis performed by two-tailed *t* test; values are the mean ± S.E.M. or back transformed mean ^1^ (95% CI) of the log data obtained from ASA24. DHA: docosahexaenoic acid; DPA: docosapentaenoic acid; EPA: eicosapentaenoic acid.

**Table 2:** Composition of select nutrients and carotenoids in 28 g of goji berries. * Lutein estimated from goji berries cultivated in Ningxia province, China (Zhao et al., 2013).

Nutrient	Amount
Calorie (Kcal)	95.1
Total Carbohydrate (g)	21.4
Fat (g)	0.4
Protein (g)	2.8
Fiber (g)	2.7
Total sugars (g)	15.1
**Carotenoids**	
Zeaxanthin (mg)	28.8
β-carotene (µg)	225
Trans β-carotene (µg)	110
α-carotene (µg)	13.8
Lycopene (µg)	<5.6
Lutein estimate * (mg)	0.15

**Table 3:** Baseline measurements of participants in the goji berry (GB) and the lutein and zeaxanthin supplement (LZ) group.

	GB Group (*n* = 13)	LZ Group (*n* = 14)	*p*-Value
Age (years)	55.9 ± 1.7	55.8 ± 1.4	0.94
Sex (F), n (%)	9 (69.2)	10 (71.4)	-
**MPOD**			
0.25 RE	0.67 ± 0.06	0.68 ± 0.06	0.88
0.5 RE	0.54 ± 0.07	0.58 ± 0.05	0.51
1 RE	0.36 ± 0.03	0.39 ± 0.03	0.32
1.75 RE	0.16 ± 0.02	0.16 ± 0.02	0.77
SC Score	369.5 ± 44.9	397.8 ± 39.6	0.64

Variables were not significantly different between the two groups. Statistical analysis was performed by two-tailed *t*-tests; data are presented as mean ± S.E.M. MPOD: macular pigment optical density; RE: retinal eccentricity degrees; SC: skin carotenoid.

## Supplementary Materials

The following are available online at https://www.mdpi.com/article/10.3390/nu13124409/s1, Table S1: Macular pigment optical density (MPOD) and skin carotenoid (SC) scores in the goji berry (GB) and lutein + zeaxanthin supplement (LZ) groups at Day 0 (SV1), Day 45 (SV2), and Day 90 (SV3). Values are the mean ± S.E.M. RE: retinal eccentricity. Figure S1.1: Positive correlations between skin carotenoid and MPOD at 0.25, 0.5, and 1 RE degrees in data from the goji berry GB and LZ groups combined. Scatter plots and 95% CI (blue shades) of the linear relationship between skin carotenoid and MPOD at 0.25 RE (a), 0.5 RE (b), 1 RE (c), and 1.75 RE (d). Figure S1.2: The skin carotenoid score and MPOD were not correlated in the GB group at any of the four RE degrees. Scatter plots and 95% CI (blue shades) of the linear relationship between skin carotenoid and MPOD at 0.25 RE (a), 0.5 RE (b), 1 RE (c), and 1.75 RE (d). Figure S1.3: Positive correlation between skin carotenoid and MPOD at 0.25, 0.5, and 1 RE degrees in the LZ group. Scatter plots and 95% CI (blue shades) of the linear relationship between skin carotenoid and MPOD at 0.25 RE (a), 0.5 RE (b), 1 RE (c), and 1.75 RE (d).

## References

[1] Heesterbeek T.J., Lorés-Motta L., Hoyng C.B., Lechanteur Y.T.E., den Hollander A.I. (2020). Risk Factors for Progression of Age-Related Macular Degeneration. Ophthalmic Physiol. Opt..

[2] World Health Organization Blindness and Vision Impairment. https://www.who.int/news-room/fact-sheets/detail/blindness-and-visual-impairment.

[3] Mitchell P., Liew G., Gopinath B., Wong T.Y. (2018). Age-Related Macular Degeneration. Lancet.

[4] Eisenhauer B., Natoli S., Liew G., Flood V.M. (2017). Lutein and Zeaxanthin—Food Sources, Bioavailability and Dietary Variety in Age-related Macular Degeneration Protection. Nutrients.

[5] Howells O., Eperjesi F., Bartlett H. (2011). Measuring Macular Pigment Optical Density in Vivo: A Review of Techniques. Graefe’s Arch. Clin. Exp. Ophthalmol..

[6] Arunkumar R., Calvo C.M., Conrady C.D., Bernstein P.S. (2018). What Do We Know about the Macular Pigment in AMD: The Past, the Present, and the Future. Eye.

[7] Rodriguez-Concepcion M., Avalos J., Bonet M.L., Boronat A., Gomez-Gomez L., Hornero-Mendez D., Limon M.C., Meléndez-Martínez A.J., Olmedilla-Alonso B., Palou A. (2018). A Global Perspective on Carotenoids: Metabolism, Biotechnology, and Benefits for Nutrition and Health. Prog. Lipid Res..

[8] Ranard K.M., Jeon S., Mohn E.S., Griffiths J.C., Johnson E.J., Erdman J.W. (2017). Dietary Guidance for Lutein: Consideration for Intake Recommendations Is Scientifically Supported. Eur. J. Nutr..

[9] Johnson E.J., Maras J.E., Rasmussen H.M., Tucker K.L. (2010). Intake of Lutein and Zeaxanthin Differ with Age, Sex, and Ethnicity. J. Am. Diet. Assoc..

[10] Mares J.A., LaRowe T.L., Snodderly D.M., Moeller S.M., Gruber M.J., Klein M.L., Wooten B.R., Johnson E.J., Chappell R.J. (2006). Predictors of Optical Density of Lutein and Zeaxanthin in Retinas of Older Women in the Carotenoids in Age-Related Eye Disease Study, an Ancillary Study of the Women’s Health Initiative. Am. J. Clin. Nutr..

[11] Carpentier S., Knaus M., Suh M. (2009). Associations between Lutein, Zeaxanthin, and Age-Related Macular Degeneration: An Overview. Crit. Rev. Food Sci. Nutr..

[12] Hernández-Zimbrón L.F., Zamora-Alvarado R., Ochoa-De La Paz L., Velez-Montoya R., Zenteno E., Gulias-Cañizo R., Quiroz-Mercado H., Gonzalez-Salinas R. (2018). Age-Related Macular Degeneration: New Paradigms for Treatment and Management of AMD. Oxid. Med. Cell. Longev..

[13] Potterat O. (2010). Goji (Lycium Barbarum and L. Chinense): Phytochemistry, Pharmacology and Safety in the Perspective of Traditional Uses and Recent Popularity. Planta Med..

[14] Widomska J., Paul Sangiovanni J., Subczynski W.K. (2020). Why Is Zeaxanthin the Most Concentrated Xanthophyll in the Central Fovea?. Nutrients.

[15] Karioti A., Bergonzi M.C., Vincieri F.F., Bilia A.R. (2014). Validated Method for the Analysis of Goji Berry, a Rich Source of Zeaxanthin Dipalmitate. J. Agric. Food Chem..

[16] Breithaupt D.E., Weller P., Wolters M., Hahn A. (2004). Comparison of Plasma Responses in Human Subjects after the Ingestion of 3R,3R′-Zeaxanthin Dipalmitate from Wolfberry (Lycium Barbarum) and Non-Esterified 3R,3R′-Zeaxanthin Using Chiral High-Performance Liquid Chromatography. Br. J. Nutr..

[17] Amagase H., Farnsworth N.R. (2011). A Review of Botanical Characteristics, Phytochemistry, Clinical Relevance in Efficacy and Safety of Lycium Barbarum Fruit (Goji). Food Res. Int..

[18] Bone R.A., Landrum J.T., Fernandez L., Tarsis S.L. (1988). Analysis of the Macular Pigment by HPLC: Retinal Distribution and Age Study. Investig. Ophthalmol. Vis. Sci..

[19] Li S., Liu N., Lin L., Sun E.D., Da Li J., Li P.K. (2018). Macular Pigment and Serum Zeaxanthin Levels with Goji Berry Supplement in Early Age-Related Macular Degeneration. Int. J. Ophthalmol..

[20] USDA FoodData Central. https://fdc.nal.usda.gov/fdc-app.html#/food-details/173032/nutrients.

[21] Jahns L., Johnson L.A.K., Conrad Z., Bukowski M., Raatz S.K., Jilcott Pitts S., Wang Y., Ermakov I.V., Gellermann W. (2019). Concurrent Validity of Skin Carotenoid Status as a Concentration Biomarker of Vegetable and Fruit Intake Compared to Multiple 24-h Recalls and Plasma Carotenoid Concentrations across One Year: A Cohort Study. Nutr. J..

[22] Rush E., Amoah I., Diep T., Jalili-Moghaddam S. (2020). Determinants and Suitability of Carotenoid Reflection Score as a Measure of Carotenoid Status. Nutrients.

[23] Pitts S.B.J., Jahns L., Wu Q., Moran N.E., Bell R.A., Truesdale K.P., Laska M.N. (2018). A Non-Invasive Assessment of Skin Carotenoid Status through Reflection Spectroscopy Is a Feasible, Reliable and Potentially Valid Measure of Fruit and Vegetable Consumption in a Diverse Community Sample. Public Health Nutr..

[24] Iannaccone A., Carboni G., Forma G., Mutolo M., Jennings B. (2016). Macular Pigment Optical Density and Measures of Macular Function: Test-Retest Variability, Cross-Sectional Correlations, and Findings from the Zeaxanthin Pilot Study of Response to Supplementation (ZEASTRESS-Pilot). Foods.

[25] Zhao L.Q., Qiu Z.Q., Narasimhamoorthy B., Greaves J.A. (2013). Development of a Rapid, High-Throughput Method for Quantification of Zeaxanthin in Chinese Wolfberry Using HPLC-DAD. Ind. Crops Prod..

[26] Bucheli P., Vidal K., Shen L., Gu Z., Zhang C., Miller L.E., Wang J. (2011). Goji Berry Effects on Macular Characteristics and Plasma Antioxidant Levels. Optom. Vis. Sci..

[27] Bovier E.R., Renzi L.M., Hammond B.R. (2014). A Double-Blind, Placebo-Controlled Study on the Effects of Lutein and Zeaxanthin on Neural Processing Speed and Efficiency. PLoS ONE.

[28] Ma L., Liu R., Du J.H., Liu T., Wu S.S., Liu X.H. (2016). Lutein, Zeaxanthin and Meso-Zeaxanthin Supplementation Associated with Macular Pigment Optical Density. Nutrients.

[29] Conrady C.D., Bell J.P., Besch B.M., Gorusupudi A., Farnsworth K., Ermakov I., Sharifzadeh M., Ermakova M., Gellermann W., Bernstein P.S. (2017). Correlations between Macular, Skin, and Serum Carotenoids. Investig. Ophthalmol. Vis. Sci..

[30] Wang C.C., Chang S.C., Inbaraj B.S., Chen B.H. (2010). Isolation of Carotenoids, Flavonoids and Polysaccharides from Lycium Barbarum L. and Evaluation of Antioxidant Activity. Food Chem..

[31] Song M.K., Salam N.K., Roufogalis B.D., Huang T.H.W. (2011). Lycium Barbarum (Goji Berry) Extracts and Its Taurine Component Inhibit PPAR-γ-Dependent Gene Transcription in Human Retinal Pigment Epithelial Cells: Possible Implications for Diabetic Retinopathy Treatment. Biochem. Pharmacol..

[32] Yossa Nzeuwa I.B., Guo B., Zhang T., Wang L., Ji Q., Xia H., Sun G. (2019). Comparative Metabolic Profiling of Lycium Fruits (*Lycium barbarum* and *Lycium chinense*) from Different Areas in China and from Nepal. J. Food Qual..

[33] Bungau S., Abdel-Daim M.M., Tit D.M., Ghanem E., Sato S., Maruyama-Inoue M., Yamane S., Kadonosono K. (2019). Health Benefits of Polyphenols and Carotenoids in Age-Related Eye Diseases. Oxid. Med. Cell. Longev..

[34] Neelam K., Dey S., Sim R., Lee J., Au Eong K.G. (2021). Fructus Lycii: A Natural Dietary Supplement for Amelioration of Retinal Diseases. Nutrients.

[35] Trieschmann M., Beatty S., Nolan J.M., Hense H.W., Heimes B., Austermann U., Fobker M., Pauleikhoff D. (2007). Changes in Macular Pigment Optical Density and Serum Concentrations of Its Constituent Carotenoids Following Supplemental Lutein and Zeaxanthin: The LUNA Study. Exp. Eye Res..

[36] Ma L., Dou H.L., Wu Y.Q., Huang Y.M., Huang Y.B., Xu X.R., Zou Z.Y., Lin X.M. (2012). Lutein and Zeaxanthin Intake and the Risk of Age-Related Macular Degeneration: A Systematic Review and Meta-Analysis. Br. J. Nutr..

[37] Chew E.Y., Clemons T.E., SanGiovanni J.P., Danis R.P., Ferris F.L., Elman M.J., Antoszyk A.N., Ruby A.J., Orth D., Bressler S.B. (2014). Secondary Analyses of the Effects of Lutein/Zeaxanthin on Age-Related Macular Degeneration Progression AREDS2 Report No. 3. JAMA Ophthalmol..

[38] Age-Related Eye Disease Study Research Group (2001). A Randomized, Placebo-Controlled, Clinical Trial of High-Dose Supplementation with Vitamins C and E, Beta Carotene, and Zinc for Age-Related Macular Degeneration and Vision Loss: AREDS Report No. 8. Arch. Ophthalmol..

[39] Chew E.Y., Clemons T.E., SanGiovanni J.P., Danis R., Ferris F.L., Elman M., Antoszyk A., Ruby A., Orth D., Bressler S. (2013). Lutein + Zeaxanthin and Omega-3 Fatty Acids for Age-Related Macular Degeneration: The Age-Related Eye Disease Study 2 (AREDS2) Randomized Clinical Trial. JAMA.

[40] Maret W., Sandstead H.H. (2006). Zinc Requirements and the Risks and Benefits of Zinc Supplementation. J. Trace Elem. Med. Biol..

[41] Chiu C.J., Chang M.L., Zhang F.F., Li T., Gensler G., Schleicher M., Taylor A. (2014). The Relationship of Major American Dietary Patterns to Age-Related Macular Degeneration. Am. J. Ophthalmol..

[42] USDA What We Eat in America, NHANES 2017–2018, Individuals 2 Years and Over. https://www.ars.usda.gov/ARSUserFiles/80400530/pdf/1718/Table_1_NIN_GEN_17.pdf.

[43] Scott T.M., Rasmussen H.M., Chen O., Johnson E.J. (2017). Avocado Consumption Increases Macular Pigment Density in Older Adults: A Randomized, Controlled Trial. Nutrients.

[44] Edwards C.G., Walk A.M., Thompson S.V., Reeser G.E., Erdman J.W., Burd N.A., Holscher H.D., Khan N.A. (2020). Effects of 12-Week Avocado Consumption on Cognitive Function among Adults with Overweight and Obesity. Int. J. Psychophysiol..

[45] Van Der Made S.M., Kelly E.R., Kijlstra A., Plat J., Berendschot T.T.J.M. (2016). Increased Macular Pigment Optical Density and Visual Acuity Following Consumption of a Buttermilk Drink Containing Lutein-Enriched Egg Yolks: A Randomized, Double-Blind, Placebo-Controlled Trial. J. Ophthalmol..

[46] Vishwanathan R., Goodrow-Kotyla E.F., Wooten B.R., Wilson T.A., Nicolosi R.J. (2009). Consumption of 2 and 4 Egg Yolks/d for 5 Wk Increases Macular Pigment Concentrations in Older Adults with Low Macular Pigment Taking Cholesterol-Lowering Statins. Am. J. Clin. Nutr..

[47] Hammond B.R., Johnson E.J., Russell R.M., Krinsky N.I., Yeum K.J., Edwards R.B., Snodderly D.M. (1997). Dietary Modification of Macular Pigment Density. Investig. Ophthalmol. Vis. Sci..

[48] Phelan D., Prado-Cabrero A., Nolan J.M. (2017). Stability of Commercially Available Macular Carotenoid Supplements in Oil and Powder Formulations. Nutrients.

[49] Obana A., Gohto Y., Nakazawa R., Moriyama T., Gellermann W., Bernstein P.S. (2020). Effect of an Antioxidant Supplement Containing High Dose Lutein and Zeaxanthin on Macular Pigment and Skin Carotenoid Levels. Sci. Rep..

