# Supplementary material for: Goji Berry Intake Increases Macular Pigment Optical Density in Healthy Adults: A Randomized Pilot Trial
Source: Nutrients. 2021 Dec 9;13(12):4409. doi: 10.3390/nu13124409 (PMC8708314; doi:10.3390/nu13124409)
Supplement: Supplementary file 1 [file nutrients-13-04409-s001.zip › nutrients-1450610-supplementary.pdf]

Supplementary Table S1. Macular pigment optical density (MPOD) and skin carotenoid (SC) scores in the goji berry (GB) and lutein + zeaxanthin supplement (LZ) groups at Day 0 (SV1), Day 45 (SV2), and Day 90 (SV3). Values are the mean ± S.E.M. RE: retinal eccentricity.

		**Day 0**	**Day 45**	**Day 90**	p within treatment			p over-all
**MPOD**					Day 0 vs. Day 45	Day 45 vs. Day 90	Day 0 vs. Day 90	
0.25 RE								
	GB	0.67 ± 0.06	0.74 ± 0.06	0.76 ± 0.06	0.16	0.42	**0.029**	0.93
	LZ	0.68 ± 0.06	0.74 ± 0.06	0.74 ± 0.06	0.11	0.88	0.14	
0.5 RE								
	GB	0.54 ± 0.07	0.55 ± 0.05	0.58 ± 0.05	0.65	0.3	0.14	0.65
	LZ	0.59 ± 0.05	0.59 ± 0.05	0.60 ± 0.05	0.83	0.71	0.54	
1 RE								
	GB	0.36 ± 0.03	0.39 ± 0.03	0.40 ± 0.03	0.2	0.51	0.06	0.74
	LZ	0.40 ± 0.03	0.41 ± 0.03	0.39 ± 0.03	0.36	0.37	0.97	
1.75 RE								
	GB	0.16 ± 0.02	0.15 ± 0.03	0.21 ± 0.03	0.75	**0.044**	**0.021**	0.99
	LZ	0.17 ± 0.02	0.17 ± 0.02	0.19 ± 0.03	0.86	0.39	0.29	
**SC**								
	GB	369.5 ± 44.9	421.4 ± 44.7	431.4 ± 44.7	**0.025**	0.56	**0.006**	0.73
	LZ	397.8 ± 39.6	442.2 ± 43.9	435.6 ± 43.8	0.17	0.7	0.3	

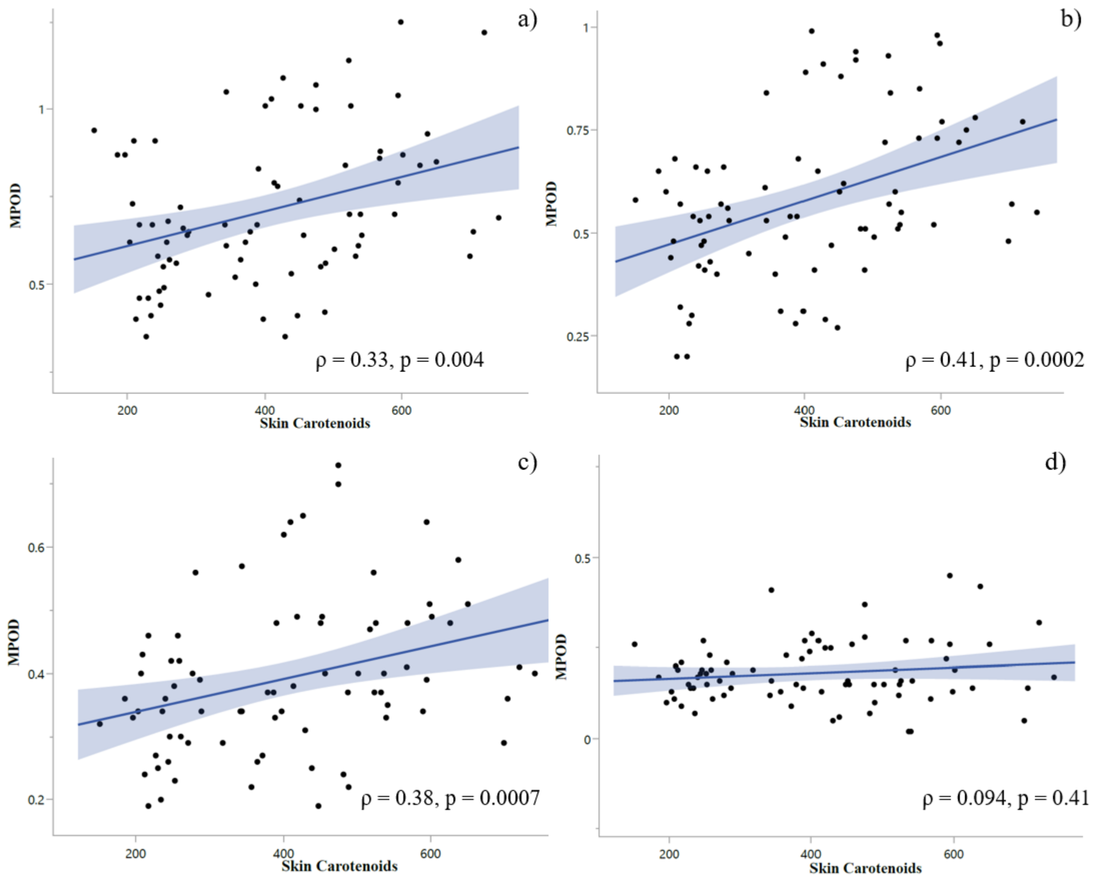

Supplementary Figure S1.1. Positive correlations between skin carotenoid and macular pigment optical density (MPOD) at 0.25, 0.5, and 1 retinal eccentricity (RE) degrees in data from the goji berry (GB) and lutein and zeaxanthin supplement (LZ) groups combined. Scatter plots and 95% CI (blue shades) of the linear relationship between skin carotenoid and MPOD at 0.25 RE (a), 0.5 RE (b), 1 RE (c), and 1.75 RE (d).

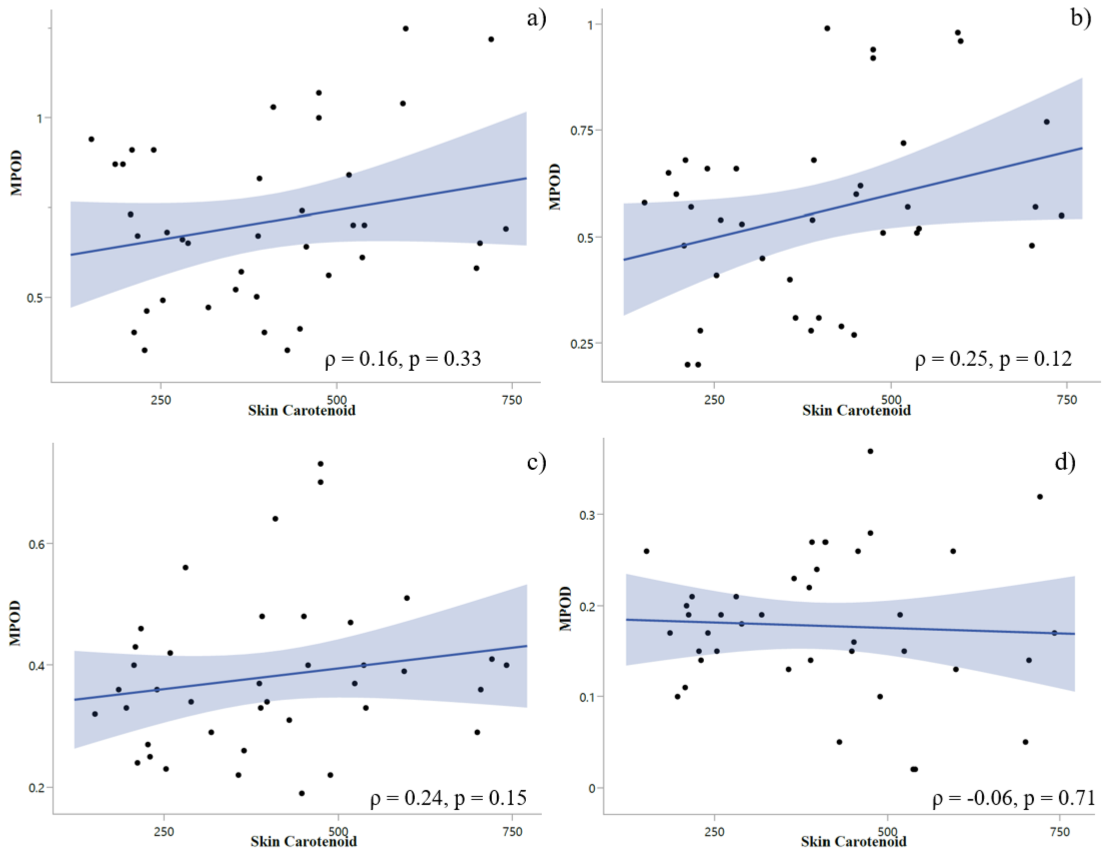

Supplementary Figure S1.2. The skin carotenoid score and macular pigment optical density (MPOD) were not correlated in the goji berry (GB) group at any of the four retinal eccentricity (RE) degrees. Scatter plots and 95% CI (blue shades) of the linear relationship between skin carotenoid and MPOD at 0.25 RE (a), 0.5 RE (b), 1 RE (c), and 1.75 RE (d).

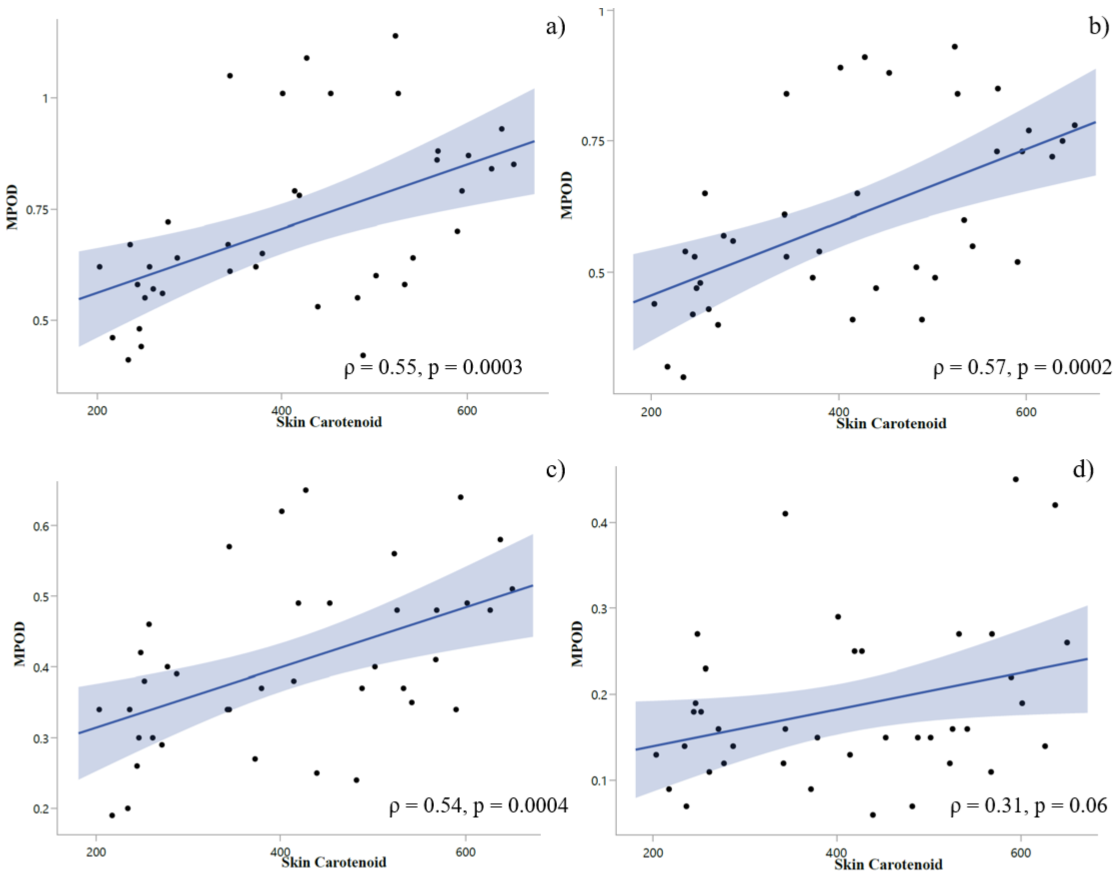

Supplementary Figure S1.3. Positive correlation between skin carotenoid and macular pigment optical density (MPOD) at 0.25, 0.5, and 1 retinal eccentricity (RE) degrees in the lutein and zeaxanthin supplement (LZ) group. Scatter plots and 95% CI (blue shades) of the linear relationship between skin carotenoid and MPOD at 0.25 RE (a), 0.5 RE (b), 1 RE (c), and 1.75 RE (d).
